# Fusion of 3D-CMR-perfusion with 3D-MR-coronary angiography

**DOI:** 10.1186/1532-429X-17-S1-P88

**Published:** 2015-02-03

**Authors:** Alexander Gotschy, Lukas Wissmann, Datta Singh Goolaub, Markus Niemann, Sebastian Kozerke, Robert Manka

**Affiliations:** Institute for Biomedical Engineering, University and ETH Zurich, Zurich, Switzerland; Department of Cardiology, University Hospital Zurich, Zurich, Switzerland

## Background

The most relevant parameters for the assessment of coronary artery disease (CAD) are myocardial perfusion and the status of the coronary arteries. It has been shown that hybrid imaging strategies to acquire both parameters such as SPECT with CT-angiography provide an added value for clinical decision making in the treatment of CAD^1^. However, SPECT and CT expose the patient to ionizing radiation and, in large prospective trials, SPECT showed inferior sensitivity to detect CAD when compared with CMR-perfusion^2^. Therefore, the aim of this study was to investigate the potential additive value of 3D-MR coronary angiography (MRCA) on a 3D-CMR perfusion protocol.

## Methods

In this study, eleven patients with suspected CAD were scheduled for an invasive X-ray coronary angiography (XA) and a CMR examination. The CMR protocol consisted of adenosine stress-rest 3D-CMR perfusion, late gadolinium enhancement (LGE) and MRCA examinations. All examinations were performed on a 3T clinical scanner. In XA, coronary stenosis ≥50% was classified as significant. In the 3D-CMR perfusion scans, the myocardial ischemic burden (MIB) was measured by determining hypoperfused areas which were not scar tissue as determined from LGE images and normalized to left-ventricular myocardial volume. MRCA scans were evaluated by an experienced reader and each vessel was graded as no/low-grade stenosis or significant stenosis. Additional 3D reconstruction and fusion of 3D-MRCA and 3D-CMR perfusion was performed.

## Results

**Figure 1:**
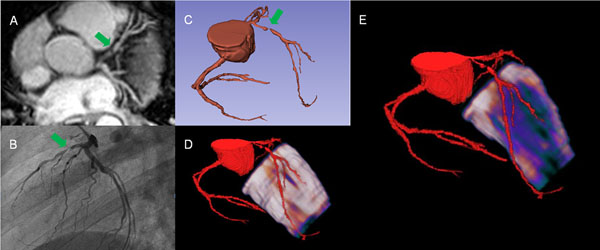
In this patient MRCA (A & C) and X-ray coronary angiography (B) reveal a high grade LAD stenosis (green arrows). 3D fusion of MRCA with CMR-perfusion shows no distinct perfusion deficit in the epicardial layer (D) but large subendocardial ischemia in the LAD territory (E).

All MIB data and 91% of the vessels in the MRCA could be evaluated successfully. CAD prevalence as defined by XA was 73% (8 of 11 patients, 16 of 33 vessels). In a vessel-based analysis, MRCA had 75% sensitivity, 79% specificity, positive predictive value of 80%, and negative predictive value of 73%. CMR-MIB/LGE measurements had 75% sensitivity, 100% specificity, positive predictive value of 100%, and negative predictive value of 81%. The combined evaluation of MRCA with CMR-MIB/LGE resulted in 94% sensitivity, 82% specificity, positive predictive value of 83%, and negative predictive value of 93%. Additional fusion of MRCA with CMR-MIB scans allowed to display the coronary anatomy in relation to the myocardial perfusion deficits (Fig. 1).

## Conclusions

For the assessment of CAD, combined evaluation of 3D-MRCA with 3D-CMR perfusion had superior sensitivity at the cost of a loss in specificity when compared to CMR-MIB/LGE alone in a vessel-based approach. The additional fusion of both modalities can provide information on the position of the coronary arteries to correlate coronary stenoses with non-distinct perfusion deficits.

## Funding

The authors acknowledge support from the Swiss National Science Foundation, Bayer Healthcare and Philips Healthcare.
